# Mitochondrial Respiration in Peripheral Blood Mononuclear Cells Negatively Correlates with Disease Severity in Pulmonary Arterial Hypertension

**DOI:** 10.3390/jcm11144132

**Published:** 2022-07-16

**Authors:** Natascha Sommer, Finn Fabian Theine, Oleg Pak, Khodr Tello, Manuel Richter, Henning Gall, Jochen Wilhelm, Rajkumar Savai, Norbert Weissmann, Werner Seeger, Hossein A. Ghofrani, Matthias Hecker

**Affiliations:** 1 Universities of Giessen and Marburg Lung Center (UGMLC), Member of the German Center for Lung Research (DZL), Excellence Cluster Cardio-Pulmonary Institute (CPI), Justus-Liebig University, 35392 Giessen, Germany; finn.theine@googlemail.com (F.F.T.); oleg.pak@innere.med.uni-giessen.de (O.P.); khodr.tello@innere.med.uni-giessen.de (K.T.); manuel.richter@innere.med.uni-giessen.de (M.R.); henning.gall@innere.med.uni-giessen.de (H.G.); jochen.wilhelm@chemie.bio.uni-giessen.de (J.W.); savai.rajkumar@innere.med.uni-giessen.de (R.S.); norbert.weissmann@innere.med.uni-giessen.de (N.W.); werner.seeger@innere.med.uni-giessen.de (W.S.); ardeschir.ghofrani@innere.med.uni-giessen.de (H.A.G.); matthias.hecker@innere.med.uni-giessen.de (M.H.); 2 Institute for Lung Health (ILH), 35392 Giessen, Germany; 3 Department of Lung Development and Remodeling, Max Planck Institute for Heart and Lung Research, 61231 Bad Nauheim, Germany; 4 Department of Medicine, Imperial College London, London W12 0NN, UK

**Keywords:** peripheral blood mononuclear cells, mitochondria, pulmonary arterial hypertension, erythroid-specific 5-aminolevulinate synthase 2

## Abstract

Mitochondrial and immune cell dysfunction contributes to the development of pulmonary arterial hypertension (PAH). We thus aimed to investigate mitochondrial respiration and mitochondrial gene expression patterns in the peripheral blood mononuclear cells (PBMC) of patients with idiopathic and hereditary PAH and their correlation to disease parameters. Mitochondrial respiration determined using high-resolution respirometry was not significantly different in PBMC when comparing an outpatient cohort of PAH patients with healthy controls. However, when directly comparing mitochondrial respiration to the hemodynamic parameters of an inpatient PAH cohort, mitochondrial respiration negatively correlated with pulmonary vascular resistance (PVR) and positively correlated with the cardiac index (CI). Furthermore, microarray analysis shows upregulation of mitochondrial erythroid-specific 5-aminolevulinate synthase 2 (ALAS2), as well as the regulation of genes involved in iron and heme metabolism, in the PBMC of patients with PAH, with ALAS2 upregulation in PAH patients being confirmed on the protein level. Multiple regression analysis with age and gender as confounders showed that both PVR and hemoglobin content negatively correlated with maximal respiration. Therefore, we conclude that mitochondrial function in the PBMC of PAH patients is affected by disease severity. However, further studies to investigate cell-type-specific alterations and functional consequences are necessary.

## 1. Introduction

Pulmonary arterial hypertension (PAH) is characterized by the abnormal remodeling and occlusion of pulmonary precapillary vessels, with a subsequent increase in pulmonary vascular resistance. Similar to cancer cells, in PAH, cellular metabolism is shifted from glucose oxidation to anaerobic glycolysis, which promotes proliferative pathways. Further mitochondrial alterations, including changes in mitochondrial dynamics (increased fission) and membrane potential (hyperpolarization), may also be important drivers of pulmonary vascular remodeling leading to PAH [1]. Previous studies have shown decreased mitochondrial function and respiration in pulmonary arterial smooth muscle and endothelial cells in animal models and human PAH [2,3,4]. Moreover, it has been suggested that the mitochondrial dysfunction underlying PAH is a systemic disease [1], and thus, easily accessible cell types such as peripheral blood mononuclear cells (PBMC) may reflect pathological alterations in pulmonary cells. Along these lines, the response of PBMC has been used in a previous study as a marker for treatment responses in PAH patients [5,6]. Furthermore, alterations in the immune system, including in peripheral immune cells, contribute to the development of PAH [7]. Previous microarray analyses have illustrated the regulation of several pathways in the PBMC of PAH patients, particularly in terms of the upregulation of genes involved in inflammatory pathways [8]. One study found the enrichment of genes for erythrocyte maturation specifically in PAH, which was correlated with hemodynamic measures of disease severity in idiopathic PAH (IPAH) patients. Interestingly, the erythroid-specific 5-aminolevulinate synthase 2 (ALAS2), which is located in mitochondria, was one of the upregulated genes in these patients [9]. The upregulation of genes encoding for different cytochrome c oxidase subunits was also observed in another set of PAH patients’ PBMC [10]. We thus aimed to characterize mitochondrial function in the PBMC of PAH patients to understand if mitochondrial alterations underlie disease development or progress. To this end, we investigated mitochondrial respiration in the PBMC of patients with hereditary PAH (HPAH) or IPAH and performed microarray analysis on these patients, specifically focusing on mitochondrial gene regulation.

## 2. Materials and Methods

### 2.1. Ethical Considerations

Patient informed consent was obtained before the study commenced. This study was approved by the local authorities (AZ 17/18) and accords with the ethical principles for medical research involving human subjects set out in the Declaration of Helsinki.

### 2.2. Patient Cohorts

We investigated two cohorts: (1) We compared the mitochondrial respiration of PBMC isolated from age- and sex-matched controls (*n* = 10) to PAH patients from our outpatient clinic (outpatient cohort, *n* = 15) to investigate alterations in mitochondrial respiration associated with the disease. (2) To correlate mitochondrial respiration with disease severity, we investigated a second inpatient group (inpatient cohort, *n* = 14) with clinical indications for right heart catheterization so that sample collection and hemodynamic assessment could be performed at the same time point. We included patients with the diagnosis of IPAH and HPAH (according to the Nice classification) to exclude the effects of underlying conditions in PAH associated with other diseases (see patients’ characteristics, Table 1). The control group included either healthy volunteers or patients with invasively excluded PAH.

### 2.3. Mitochondrial Respiration

Mitochondrial respiration was determined using high-resolution respirometry in the presence or absence of different mitochondrial inhibitors to analyze basal respiration, electron leak respiration, and maximal respiratory capacity, the latter via step-wise titration of FCCP to precisely determine maximal respiration [11].

### 2.4. Microarray

Microarray analysis was performed to detect gene regulation patterns in PAH patients (For details, please see online Supplementary Materials).

### 2.5. Western Blot

Western blot analysis was performed in lung lysates from donor and IPAH patients for the quantification of ALAS2 protein (H00000212-M01, Novus Bio Science Reagents, Centennial, CO, USA). The level expression of β-actin (ACTB, A2228, Sigma-Aldrich, Burlington, VT, USA) protein was used as the loading control. The dilution of primary antibodies was 1:1000 for ALAS2 and 1:50,000 for β-actin. The dilution of corresponding secondary antibodies was 1:5000. The incubation time for primary antibodies was overnight at 4 °C, while for corresponding secondary antibodies, it was 1h at room temperature.

### 2.6. Statistical Analysis

Statistical analysis was performed with Jamovi (Version 1.6.23.0) using ANCOVA for the comparison of respiratory states in the patient groups (with age and gender as covariates), as well as GraphPad Prism 9 using a linear model for correlation analysis and multiple regression analysis. For Detailed Methods, please Refer to the Supplementary Methods.

## 3. Results

**Figure 1:**
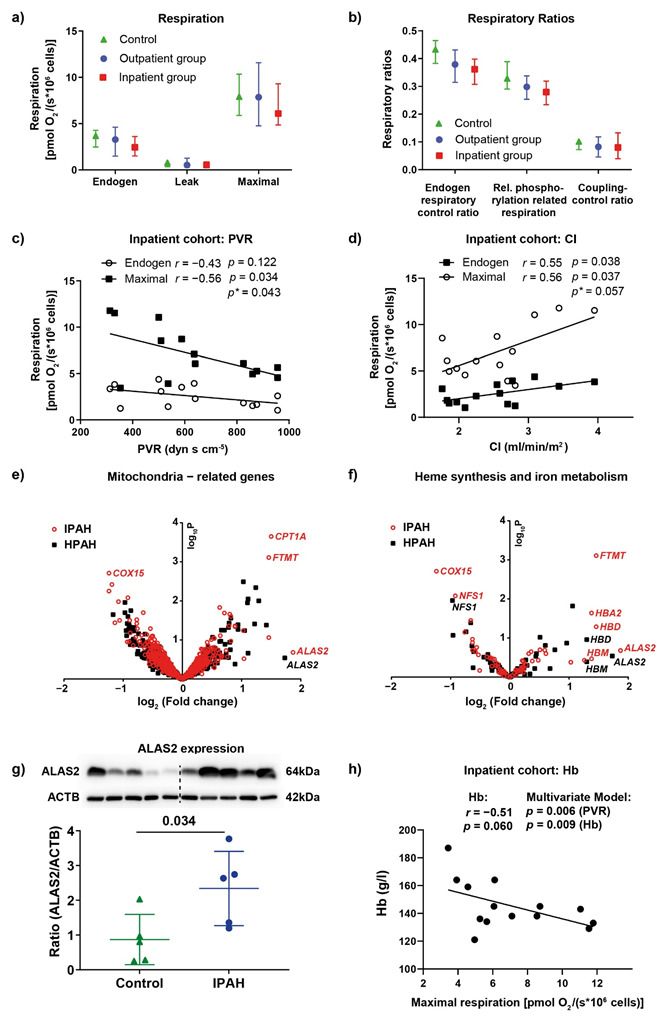
Mitochondrial respiration and its correlation with PAH. (**a**) Mitochondrial respiration in the intact, unstimulated PBMC of control patients (*n* = 10), outpatients (*n* = 15), and inpatients (*n* = 14). Mitochondrial respiration was determined as oxygen consumption in unstimulated PBMC (“endogen”) after the inhibition of ATPase using oligomycin (representing proton leak respiration, “leak”) and after maximal stimulation via step-wise titration with the uncoupler FCCP (“maximal”). (**b**) Respiratory control ratios representing the phosphorylation control ratio (endogen/maximal: limitation of OXPHOS capacity by the phosphorylation system), relative OXPHOS capacity (endogen-leak/maximal), and the coupling control ratio (oligo/maximal: index for uncoupling). Data are shown as the median and interquartile range. (**c**,**d**) The correlation between endogen and the maximal respiration of PBMC from the inpatient cohort with PVR: (**c**) the CI; (**d**) determined using linear regression analysis (*p*- and *r*-values). Multivariate analysis for the adjustment of maximal respiration for age and gender (p*-values). (**e**,**f**) Microarray analysis comparing the gene expression of the PBMC of control patients (*n* = 7) with that of HPAH *(n =* 3) and IPAH (*n* = 4) patients. The volcano plots show mitochondria-related genes: (**e**) genes related to heme synthesis and iron metabolism; (**f**) COX15: Cytochrome c oxidase assembly protein COX15 homolog, also known as heme A synthase, NFS1: cysteine desulfurase, ALAS2: 5-aminolevulinate synthase 2, CPT1: carnitine-palmitoyl-transferase 1, FTMT: mitochondrial ferritin, HB: hemoglobin. (**g**) The protein expression of ALAS2; data are shown as mean ± SD, *p* = 0.034, analyzed using a *t*-test. (**h**) The correlation between the maximal respiration of the PBMC of the inpatient group and Hb determined using linear regression analysis. Multiple linear regression with maximal respiration as the dependent variable and age, gender, PVR, and Hb as the independent variables.

The mitochondrial respiration and respiratory ratios were not significantly different in PAH patients and controls (Figure 1a,b). To further investigate the relationship between mitochondrial respiration and PAH, we correlated mitochondrial respiration directly with invasively determined hemodynamics in the inpatient cohort and found a negative correlation between mitochondrial respiration and pulmonary vascular resistance (PVR) and a positive correlation with the cardiac index (CI), also after adjustment for age and gender as potential confounding factors (Figure 1c,d). To further investigate the mechanisms of decreased respiration in severe PAH, we performed microarray analysis of PBMC selected from patients with severe HPAH and IPAH (defined by a PVR above the median). As previously described for IPAH patients [9], we found upregulation of ALAS2, but with high interindividual variability. Moreover, mitochondrial ferritin (FTMT) and carnitine palmitoyltransferase I were raised in IPAH PBMC (Figure 1e). In contrast, cytochrome c oxidase assembly protein homolog (COX15; also known as heme A synthase) was downregulated in IPAH (Figure 1e). Since ALAS2, FTMT, and COX15 affect heme metabolism, we focused on the gene pathways involved in heme and iron metabolism and found genes regulating hemoglobin (Hb) synthesis to be upregulated, while genes regulating the synthesis of mitochondrial heme A (COX10) and iron–sulfur cluster proteins (NFS1, nitrogen fixation 1 or cysteine desulfurase) were downregulated (Figure 1f). We confirmed ALAS2 upregulation in the PBMC of IPAH patients on the protein level (Figure 1g). Since the key steps of heme metabolism occur in the mitochondria, we analyzed the correlation between Hb levels and mitochondrial respiration (Figure 1h). Multiple regression analysis with age and gender as confounders showed that both PVR and Hb reduced the residual variance in maximal respiration and negatively correlated with maximal respiration, which is consistent with the hypothesis that decreased mitochondrial respiration in the PBMC of PAH patients is caused by independent variations in PVR and Hb.

## 4. Discussion

To our knowledge, this is the first study to investigate mitochondrial respiration in the PBMC of PAH patients. We did not find a significant difference in mitochondrial respiration between a control group and two cohorts of IPAH/HPAH patients. However, our data provide evidence that mitochondrial respiration is decreased depending on disease severity in PAH patients, suggesting that multiple factors affect mitochondrial respiration in PAH. Since mitochondrial respiration decreased with increasing PVR, our findings accord with the concept of the metabolic switch in PAH, which includes decreased mitochondrial oxidative phosphorylation [2,3,4,12]. However, in PBMC, this may be related to other systemic factors. One factor affecting mitochondrial respiration in the PBMC of PAH patients may be the disturbance in iron metabolism and mitochondrial proteins containing iron–sulfur clusters [12,13], as we found the mRNA regulation of ALAS2, the key enzyme in the heme synthesis of erythroid progenitor cells, the regulation of various Hbs, and the regulation of the mitochondrially localized iron-sequestering protein FTMT. Additionally, the upregulation of ALAS2 in the PBMC of IPAH patients has been previously reported and accounted for the increased red blood cell precursors due to hypoxic conditions [9]. Moreover, a disturbance in the erythroid progenitor cell compartment has been shown to contribute to PH development [14]. Alternatively, non-erythroid cells may express ALAS2 under stress conditions, as previously shown in, for example, the pulmonary arteries of an animal model of PH [15]. Increased ALAS2 mRNA expression and heme metabolism were also found in the lung tissue of end-stage PAH patients [16]. Thus, alterations in heme synthesis may have affected mitochondrial respiration in our patient cohort, a hypothesis that is supported by our finding of a correlation between mitochondrial respiration and Hb levels, even after adjusting for PVR as a confounding factor. In this regard, it was shown that heme synthesis and export inhibit oxidative phosphorylation in proliferating cells [17]. Furthermore, the redistribution of iron from iron–sulfur cluster generation to heme production may further affect mitochondrial respiration, as decreased levels of iron–sulfur cluster proteins have been found in PAH [13]. Other factors, such as fatty acid oxidation (FAO), may also affect the mitochondrial respiration of PBMC in PAH (albeit through increasing respiration), as the mRNA expression of CPT1 was increased in the PBMC of IPAH patients in our microarray analysis. The role of FAO in the development of PAH has been described recently [12], and it has been shown to be a potential factor in the increase in the respiratory capacity of platelets in PAH patients [18].

## 5. Limitations of the Study

This study was conducted as a single-center study with limited *n*-numbers that did not allow for analysis of the differences between IPAH and HPAH patients. Additionally, alterations detected in the microarray analysis were confirmed for the key enzyme of hemoglobin metabolism, ALAS2, but not specifically for other genes. Therefore, in future studies, a larger patient cohort should be investigated to confirm our findings and allow for a detailed analysis of different PAH subgroups.

## 6. Conclusions

Mitochondrial respiration in the PBMC of PAH patients correlates with disease severity and Hb levels. Further studies should investigate the contribution of FAO, mitochondrial biogenesis, and mitochondrial dynamics to alterations in mitochondrial function in PAH-PBMC, as well as their consequences for PBMC function.

## Figures and Tables

**Table 1:** Patients’ characteristics. No statistically significant differences were found between the groups.

	Control *n* = 10			PAH Outpatient *n* = 15			PAH Inpatient *n* = 14		
Male %	40			27			50		
	Median	Q1	Q3	Median	Q1	Q3	Median	Q1	Q3
Age	56	28	68	59	49	64	59	50	70
Endogen	3.70	2.47	4.28	3.28	1.50	4.62	2.44	1.41	3.61
Leak	0.79	0.36	1.09	0.53	0.29	1.27	0.53	0.32	0.87
FCCP	7.92	5.89	10.35	7.86	4.76	11.58	6.09	4.86	9.30
Endogen/FCCP	0.43	0.38	0.46	0.38	0.31	0.43	0.36	0.30	0.40
Endogen-olig/FCCP	0.33	0.29	0.39	0.30	0.25	0.34	0.27	0.23	0.32
IL6 (pg/mL)	1.4	0.00	18.03	0.0	0.00	0.00	1.3	0.00	54.53
IL8 (pg/mL)	6.7	2.55	19.78	4.0	2.50	5.40	6.5	4.43	54.60
CVP (mmHg)				3.5	2.00	5.25	7.0	5.50	10.00
mPAP (mmHg)				41	27.25	50.00	46	36.00	52.25
PAWP (mmHg)				7	4.00	10.00	11	8.00	13.50
CI (l/min/m^2^)				2.8	2.52	3.42	2.6	2.03	2.88
PVR (dyn s cm^−5^)				532	313	739	562	427	834
SVR (dyn s cm^−5^)				1065	842	1231	1245	980	1764
pO_2_ (mmHg)				68	62	76	64	55	71
pCO_2_ (mmHg)				36	31	38	31	28	34
Hb (g/L)				143	132	155	141	132	160
Leucocyte (10^9^/L)				7.2	5.5	8.3	7.5	5.7	9.5
Thrombocyte (10^9^/L)				246	189	276	203	152	241
ALT (U/L)				16	12	22	17	13	25
AST (U/L)				17	13	29	21	16	24
GGT (U/L)				23	13	37	26	17	34
Total bilirubin (mg/dL)				0.6	0.0	1.0	0.8	0.6	1.1
Creatinine (mg/dL)				0.9	0.70	1.2	1.0	0.8	1.1
Urea (mg/dL)				33	24	43	29	21	43
Uric acid (mg/dL)				5.7	4.3	6.40	6.8	6.00	7.90
BNP (pg/mL)				37	14	128	107	45	193
CRP (mg/dL)				2.6	0.8	13.2	2.7	0.6	10.8
sPAP (mmHg)				45	32	78	82	66	86
TAPSE (mm)				22	20	23	21	19	24
RA (cm^2^)				13	10	15	15	12	17
Therapy									
single	—			2 *			5		
double	—			6			4		
triple	—			5			5		

Abbreviations: Respiratory measurements: Endogen: endogen respiration, Leak: leak respiration, FCCP: carbonyl cyanide-p-trifluoromethoxyphenylhydrazone; serum parameters: IL: interleukin; Invasive hemodynamics: CVP: central venous pressure, mPAP: mean pulmonary arterial pressure, PAWP: pulmonary arterial wedge pressure, CI: cardiac index, PVR: pulmonary vascular resistance, SVR: systemic vascular resistance; blood gases during right heart catheterization: pO_2_: arterial partial pressure of oxygen, pCO_2_: arterial partial pressure of carbon dioxide; routine laboratory parameters: Hb: hemoglobin, ALT: alanine aminotransferase, AST: aspartate aminotransferase, GGT: gamma-glutamyl transferase (mmHg), BNP: brain natriuretic peptide, CRP: c-reactive protein; echocardiography: sPAP: systolic pulmonary arterial pressure, TAPSE: tricuspid annular plane systolic excursion, RA: right atrium, Q1: first quartile, Q3: third quartile. * plus two patients that were on calcium channel blockers.

## Supplementary Materials

The following supporting information can be downloaded at: https://www.mdpi.com/article/10.3390/jcm11144132/s1. Online Data Supplement [5,11,19,20,21,22,23].
