# Supplementary material for: Mitochondrial Respiration in Peripheral Blood Mononuclear Cells Negatively Correlates with Disease Severity in Pulmonary Arterial Hypertension
Source: J Clin Med. 2022 Jul 16;11(14):4132. doi: 10.3390/jcm11144132 (PMC9319555; doi:10.3390/jcm11144132)
Supplement: Supplementary file 1 [file jcm-11-04132-s001.zip › jcm-1774006-supplementary.pdf]

Online Data Supplement

Mitochondrial respiration in peripheral blood mononuclear cells negatively correlates with disease severity in pulmonary arterial hypertension

by Natascha Sommer, Finn Fabian Theine, Oleg Pak, Khodr Tello, Manuel Richter, Henning Gall, Jochen Wilhelm, Rajkumar Savai, Norbert Weissmann, Werner Seeger, Hossein A. Ghofrani, Matthias Hecker

Materials and Methods:

Blood collection and PBMC isolation

The study was approved by the institutional review board (Ethikkommission des Fachbereichs Medizin, Justus-Liebig University Giessen) and written informed consent was obtained from each healthy volunteer and patient (AZ 17/18). Samples were collected between October 2018 and April 2019 at the University Hospital Giessen. Diagnosis of idiopathic pulmonary arterial hypertension (IPAH) and hereditary pulmonary arterial hypertension (HPAH) was performed according to the current guidelines during that time period [19]. The patients from the outpatient group had undergone right heart catheterization for diagnosis of PH at any time before blood sampling, the patients of the inpatient group at the same day as blood sampling. All other measurements (echocardiography, laboratory testing) were performed at the day of sampling. Patients were excluded if there was evidence of other active disease processes unrelated to PAH (e.g. systemic infection). Healthy volunteers were excluded if they reported any cardiopulmonary disease. Three control subjects were recruited from patients with suspected chronic thromboembolic pulmonary hypertension but exclusion of the disease. PBMC were isolated as described previously [5]. To isolate human PBMC, peripheral blood (15 ml) was collected by venepuncture into EDTA (ethylenediaminetetraacetic acid)-buffered collection tubes (Sarstedt, Nürnberg, Germany) and subsequently subjected to a Ficoll-Hypaque (Sigma-Aldrich, Darmstadt, Germany) gradient following the manufacturer's protocol (centrifugation for 15 min at 12.000g). All specimens were processed within 45 min of sampling.

Measurement of mitochondrial respiration

Mitochondrial respiration in PBMC was measured using a high-resolution respirometer (Oxygraph-2k; Oroboros Instruments, Innsbruck, Austria) set to 37 °C with a stirring speed of 750 r.p.m. Measurements were performed with 2 million cells per ml in RPMI 1640 (Sigma-Aldrich, Darmstadt, Germany). Respiration measurement were performed as described previously [11]. Manual titration of inhibitors and uncouplers was performed using Hamilton syringes (Hamilton Company, Reno, NV, USA). After equilibration of PBMC in the

measurement chamber different respiratory control states were determined: First, unstimulated respiration was determined (endogen respiration), followed by titration of 1µl oligomycin (Sigma-Aldrich, final concentration of 2.5 µM) to inhibit ATPase (leak respiration, representing residual oxygen consumption compensating for the proton leak, proton slippage and cation cycling across the inner mitochondrial membrane). Next, maximal respiration was induced by step-wise titration of 1µl FCCP (Sigma-Aldrich, each step reaching a final concentration of 1µM) to induced a proton leak which abolishes the inhibitory effect of the proton gradient on electron flow (maximal capacity of the respiratory chain that is not limited by the proton gradient which is determined under physiological conditions mainly by enzyme activity of the ATPase). As high concentrations of FCCP have an inhibitory effect on respiration, FCCP needs to be titrated step-wise to achieve optimal stimulatory conditions. Finally, non-mitochondrial oxygen consumption was determined by titration of 1µM Antimycin A (Sigma-Aldrich, final concentration 2µM) and subtracted from endogen, leak and maximal respiration. The course of the experiment and titration steps is provided in Supplemental figure 1. Different respiratory flux control ratios were determined by normalizing oxygen consumption at different respiratory control states to oxygen consumption at maximal respiration to obtain coupling and substrate control independent from different maximal respiration of the control and PAH groups. The following ratios were determined: 1) Endogen respiratory control ratio: endogen oxygen consumption divided by maximal oxygen consumption. Provides information on the limitation of respiratory capacity by the phosphorylation system, e.g. by ADP-dependent regulation of the ATPase. It is therefore affected by cellular demand of mitochondrial ATP, but also uncoupling or decreased maximal capacity. 2) Relative phosphorylation related respiration: Difference of endogen and leak oxygen consumption divided by maximal oxygen consumption. Provides information on the fraction of respiration that is used for ATP production. 3) Coupling control ratio: leak oxygen consumption divided by maximal oxygen consumption. Provides information on relative uncoupling.

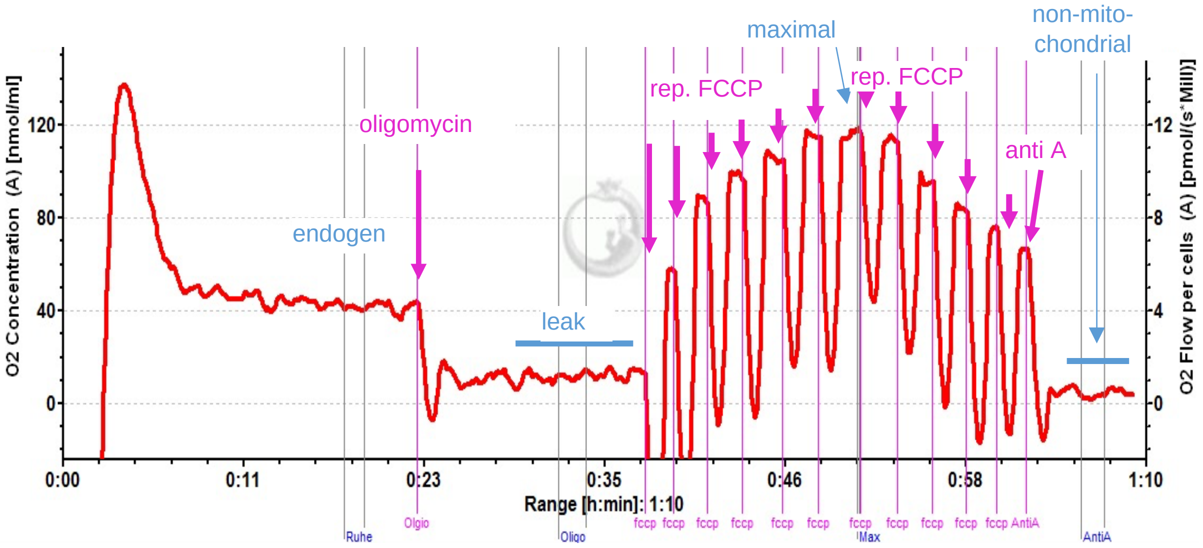

Supplemental Figure S1: Original recording of a respiration measurement of PBMC. Blue: respiratory control states. Pink: application of the inhibitors oligomycin and antimycin A (anti A) and the uncoupler carbonyl cyanide-p-trifluoromethoxyphenylhydrazone in a repetitive manner (rep. FCCP).

Microarray experiments

PBMC from a total of 7 IPAH and HPAH patients and seven controls were chosen for whole genome gene expression using oligonucleotide spotted microarray slides (Agilent Technologies). RNA was isolated using the RNeasy Mini Kit (Qiagen) following the manufacturer's instructions. Purified total RNA was amplified and Cy3-labeled using the LIRAK kit following the kit instructions. 200ng of total RNA was used per reaction. The Cy-labeled aRNA was hybridized overnight to 8x60K 60mer oligonucleotide spotted microarray slides (Agilent whole human genome microarray v3, design ID 072363). Hybridization and subsequent washing and drying of the slides were performed following the Agilent hybridization protocol. The dried slides were scanned at 2 µm/pixel resolution using the InnoScan 900 (Innopsys, Carbonne, France). Image analysis was performed with Mapix 8.5.0 software, and calculated values for all spots were saved as GenePix results files.

The data were evaluated using the R software version 3.5.1 [20] and the limma package version 2.14 [21] from BioConductor [22]. Log mean spot signals were taken for further analysis. Data was background corrected using the NormExp procedure on the negative control spots and quantile-normalized before averaging [23]. Genes were ranked for differential expression using a moderated t-statistic. Pathway analyses were done using gene set tests on the ranks of the t-values.

Western Blot analysis

PBMC were homogenized in 200 µl of Cell Lysis Buffer (Cell Signaling Technology) containing 1 mM PMSF. After 15 minutes incubation on ice, samples were centrifuged (14,000xg for 10 minutes at 4°C). Protein concentration was determined by a spectrophotometric assay (BCA assay, Pierce, Rockford, IL, USA). Twenty µg/µl of protein sample was used for Western blotting. Samples were run on a 12% sodium dodecyl sulphate-polyacrylamide gel, following transfer to a polyvinylidene fluoride membrane (PVDF; ImmobilonTM-P, Millipore Corporation, Billerica, MA, USA). Membranes were blocked in 5% non-fat dry milk in phosphate buffered saline (PBS) with 0.1% Tween 20 (PBS-T buffer). The following antibodies were applied over night at 4°C: anti-ALAS2 (H00000212-M01, Novus Bio Science Reagents, 1:1.000 diluted), and human anti-β-actin (A2228, Sigma-Aldrich; 1:50,000 diluted). After washing 4 times for 10 minutes with PBS-T buffer, the membranes were incubated for 1 hour with horseradish-peroxidase–labeled secondary antibodies (anti-rabbit W4021 and anti-mouse W4011, Promega, Madison, WI, USA; 1:5,000 diluted). Afterwards, the membranes were washed 3 times 10 minutes with PBS-T buffer. Proteins were detected by Clarity™ Western ECL Blotting Substrate (Bio-Rad).

Statistical analysis

Statistical analysis was performed with Jamovi, (Version 1.6.23.0) using ANCOVA for comparison of respiratory states in the patient groups (with age and gender as covariates), and GraphPad Prism 9 using a linear model for correlation analysis and multiple regression analysis. Analysis of pulmonary vascular resistance (PVR) and cardiac index (CI) were perfomed with log(10) transformed values.

References:

5. Sommer, N.; Droege, F.; Gamen, K.E.; Geisthoff, U.; Gall, H.; Tello, K.; Richter, M.J.; Deubner, L.M.; Schmiedel, R.; Hecker, M.; et al. Treatment with low-dose tacrolimus inhibits bleeding complications in a patient with hereditary hemorrhagic telangiectasia and pulmonary arterial hypertension. *Pulm. Circ.* **2019**, *9*, 2045894018805406. https://doi.org/10.1177/2045894018805406.
11. Hecker, M.; Sommer, N.; Mayer, K. Assessment of Short- and Medium-Chain Fatty Acids on Mitochondrial Function in Severe Inflammation. *Methods Mol. Biol.* **2021**, *2277*, 125–132. https://doi.org/10.1007/978-1-0716-1270-5_8.
19. Galie, N.; Humbert, M.; Vachiery, J.L.; Gibbs, S.; Lang, I.; Torbicki, A.; Simonneau, G.; Peacock, A.; Vonk Noordegraaf, A.; Beghetti, M.; et al. 2015 ESC/ERS Guidelines for the diagnosis and treatment of pulmonary hypertension: The Joint Task Force for the Diagnosis and Treatment of Pulmonary Hypertension of the European Society of Cardiology (ESC) and the European Respiratory Society (ERS): Endorsed by: Association for European Paediatric and Congenital Cardiology (AEPC), International Society for Heart and Lung Transplantation (ISHLT). *Eur. Respir. J.* **2015**, *46*, 903–975. https://doi.org/10.1183/13993003.01032-2015.
20. R Development Core Team. *R: A Language and Environment for Statistical Computing*; R Foundation for Statistical Computing: Vienna, Austria, 2007; ISBN 3-900051-07-0.
21. Gentleman, R.; Carey, V.J.; Huber, W.; Irizarry, R.A.; Dudoit, S. (Eds.) Limma: Linear models for microarray data. In *Bioinformatics and Computational Biology Solutions Using R and Bioconductor*; Springer: New York, NY, USA, 2005; pp. 397–420.
22. Gentleman, R.C.; Carey, V.J.; Bates, D.M.; Bolstad, B.; Dettling, M.; Dudoit, S.; Ellis, B.; Gautier, L.; Ge, Y.; Gentry, J.; et al. Bioconductor: Open software development for computational biology and bioinformatics. *Genome Biol.* **2004**, *5*, R80. https://doi.org/10.1186/gb-2004-5-10-r80.

23. Silver, J.D.; Ritchie, M.E.; Smyth, G.K. Microarray background correction: maximum likelihood estimation for the normal-exponential convolution. *Biostatistics* **2009**, *10*, 352–363. https://doi.org/10.1093/biostatistics/kxn042.
